# Targeting Hsp90 in urothelial carcinoma

**DOI:** 10.18632/oncotarget.3502

**Published:** 2015-03-26

**Authors:** Mahmoud Chehab, Tiffany Caza, Kamil Skotnicki, Steve Landas, Gennady Bratslavsky, Mehdi Mollapour, Dimitra Bourboulia

**Affiliations:** ^1^ Department of Urology, SUNY Upstate Medical University, Syracuse, NY 13210, USA; ^2^ Department of Pathology, SUNY Upstate Medical University, Syracuse, NY 13210, USA; ^3^ Upstate Cancer Research Institute, SUNY Upstate Medical University, Syracuse, NY 13210, USA; ^4^ Department of Biochemistry and Molecular Biology, SUNY Upstate Medical University, Syracuse, NY 13210, USA

**Keywords:** urothelial carcinoma, pathogenesis, bladder cancer treatments, heat shock protein-90, Hsp90 inhibitors

## Abstract

Urothelial carcinoma, or transitional cell carcinoma, is the most common urologic malignancy that carries significant morbidity, mortality, recurrence risk and associated health care costs. Despite use of current chemotherapies and immunotherapies, long-term remission in patients with muscle-invasive or metastatic disease remains low, and disease recurrence is common. The molecular chaperone Heat Shock Protein-90 (Hsp90) may offer an ideal treatment target, as it is a critical signaling hub in urothelial carcinoma pathogenesis and potentiates chemoradiation. Preclinical testing with Hsp90 inhibitors has demonstrated reduced proliferation, enhanced apoptosis and synergism with chemotherapies and radiation. Despite promising preclinical data, clinical trials utilizing Hsp90 inhibitors for other malignancies had modest efficacy. Therefore, we propose that Hsp90 inhibition would best serve as an adjuvant treatment in advanced muscle-invasive or metastatic bladder cancers to potentiate other therapies. An overview of bladder cancer biology, current treatments, molecular targeted therapies, and the role for Hsp90 inhibitors in the treatment of urothelial carcinoma is the focus of this review.

## INTRODUCTION

### 

#### Epidemiology

Bladder cancer is the fifth most common type of cancer and the second most frequent urologic malignancy in males after prostate cancer. It is the ninth leading cause of cancer death in the United States. The median age at diagnosis and death are 73 and 79 years respectively [1]. In 2014, there were an estimated 74, 690 new cases of bladder cancer and 15, 580 deaths [1]. In the United States, the estimated lifetime risk of urothelial carcinoma is 1 of 25 in men and 1 of 80 in women [2]. In fact, there is a 3:1 male-to-female predominance in urothelial carcinoma, likely related to protective effect of estrogens and increased androgen-receptor signaling in males [3–5]. Health care costs associated with treatment and surveillance of bladder cancer exceed that of all other malignancies, making the design of effective therapies essential not only for patients, but also for public health [6].

More than 90 percent of bladder cancers are urothelial carcinomas (transitional cell carcinomas). Squamous cell carcinomas comprise approximately 5 percent of bladder cancers, and less common neoplasms include micropapillary urothelial carcinoma, small cell carcinoma, sarcomas, and other rare tumors [7]. In addition, other solid tumors may metastasize to the bladder through local spread (prostate, testicular, ovarian, cervical, and endometrial cancers) [7, 8].

Of all newly diagnosed urothelial carcinomas, nearly 70 percent are non-muscle invasive and are treated surgically with transurethral resection with or without intravesical therapies [9, 10]. Non-muscle invasive urothelial carcinoma frequently recurs, in 50 to 70 percent of patients, while muscle-invasive disease has a propensity to metastasize [11]. There is a risk of progression to muscle invasive disease, occurring in up to 15 percent of non-muscle invasive tumors [11].

#### Risk factors

Advanced age and smoking are major risk factors for developing bladder cancer. A meta-analysis of epidemiologic studies showed that cigarette smokers have a four-fold increased risk than that of nonsmokers and the number of pack-years further increased that risk. More than 50 percent of urothelial carcinomas occur in smokers, which make up 14.5 to 21 percent of the population [12]. This equates to one-half of male urinary tract cancers and one-third of female urinary tract cancers that are attributed to cigarette smoking [13, 14]. Smoking promotes progression of non-invasive to muscle-invasive tumors, and cessation improves long-term prognosis when compared to chronic smokers [15]. Additional risk factors in urothelial carcinoma include chronic exposure to aromatic amine and aniline dyes, *Schistosomiasis* infection, prior pelvic irradiation, arsenic exposure, phenacetin-containing analgesics and chemotherapy drugs (particularly alkylating agents) [16].

#### Pathology

Bladder cancers are staged and prognosticated according to the tumor-node-metastasis (TNM) staging system [7]. Non-muscle invasive bladder cancers and muscle-invasive bladder cancers have distinct phenotypic, etiologic, and prognostic characteristics. Non-muscle invasive bladder cancers are, by definition, confined to the mucosa or submucosa, while muscle invasive bladder cancers invade into the muscularis propria or serosal surface of the bladder. Non-muscle invasive urothelial carcinoma develops with hyperplasia of the epithelium with development of branching vessels to form a papillary pattern [17]. Urothelial hyperplasia can progress to form low-grade urothelial carcinoma, which has a high recurrence risk, or can progress to a high-grade tumor [18]. Muscle invasive urothelial carcinoma involves dysplasia of the urothelium and occasionally progresses from carcinoma *in situ* (CIS) [17]. CIS is high grade, and has the propensity to progress to an invasive carcinoma, and muscle invasive tumors with a higher risk of metastasis [7].

#### Urothelial carcinoma pathogenesis

**Figure 1:**
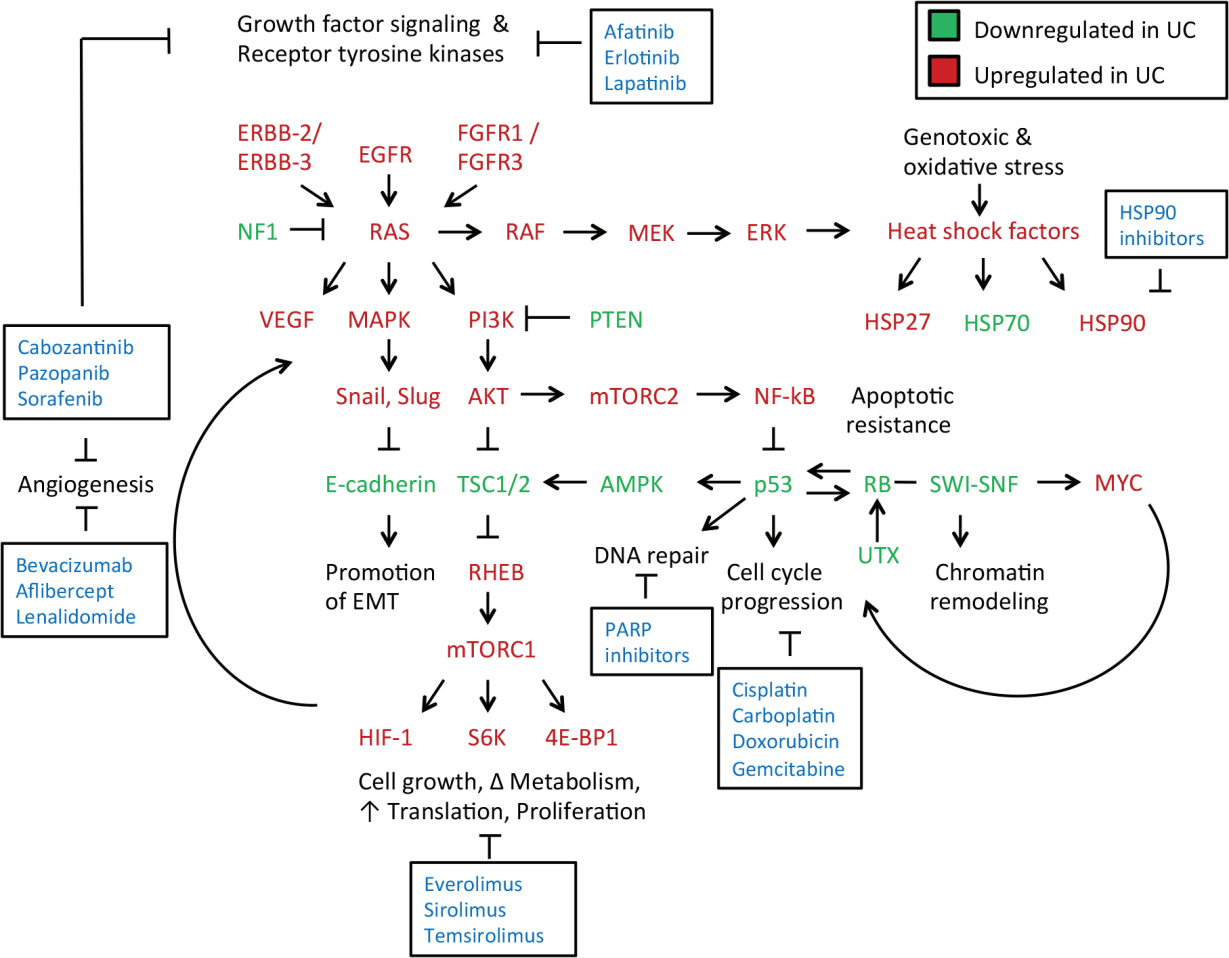
Signaling networks and treatment targets in muscle-invasive and metastatic urothelial carcinomas Growth factor signaling is increased in urothelial carcinoma [60]. This results in triggering of growth factor receptors (ERBB-2, ERBB-3, EGFR, FGFR1, FGFR3) leading to Ras activation. Hyperactivation of Ras is a key transition from a non-invasive to an invasive phenotype in urothelial carcinomas [18]. Ras hyperactivation results in phosphotidylinositol-3-kinase (PI3K) signaling, that leads to Akt and mTOR activation downstream. Ras hyperactivation also increases activity of MAP kinases, which activate key regulators of the epithelial-mesenchymal transition [81]. This ultimately leads to an inhibition of E-cadherin expression, promoting local invasion of the tumor through a loss of appropriate cell-cell adhesion [189]. Ras also induces RAF-MEK-ERK signaling, which impacts cytoskeletal dynamics as well as induces a heat shock factor response with increased activity of Hsp27 and Hsp90, as well as other components [155]. Ras is negatively regulated by NF1, which is deficient in some urothelial carcinomas, allowing for uninhibited Ras activation. PI3K activity is inhibited by PTEN, which is also deficient in some urothelial carcinomas due to mutation, leading to increased activation of Akt by PI3K [60, 190]. Akt inhibits the tuberous sclerosis complex (TSC) that acts as a negative regulator of mTORC1 activity. PI3K-Akt activation, as well as mutation within a TSC component (TSC1 or TSC2), leads to inappropriate mTORC1 activation by Rheb GTPase [191]. mTORC1 promotes numerous anabolic processes, including cell growth, metabolism, protein translation, and hypoxic signaling through increased production of hypoxia-inducible factor-1 (HIF-1) [192]. HIF-1 and vascular endothelial growth factor (VEGF) promote angiogenesis and support an intratumor vasculature. Akt also stimulates the mechanistic target of rapamycin (mTOR) complex 2 to activate NF-kB and promote cytoskeletal growth [193]. NF-kB in turn inhibits p53, which promotes apoptotic resistance [194]. Loss of p53 expression leads to uninhibited cell cycle progression, as does loss of the retinoblastoma (RB1) tumor suppressor gene [195]. Reduced RB1 expression results from mutation of its locus as well as through reduced accessibility of chromatin to transcribe its locus from inactivation of the SWI-SNF chromatin remodeling complex [84]. Increased cell cycle progression, paired with an increase in anabolic processes, promotes survival and growth of the tumor. *Molecules in red are upregulated in urothelial carcinomas, while those in green are downregulated. Molecular targeted therapies to disrupt these key processes implicated in urothelial carcinomas growth and progression are highlighted in boxes.

The molecular pathogenesis of urothelial carcinomas requires deregulation of multiple signal transduction pathways, therefore, it is a malignancy in which molecular targeted therapies will be useful to block key signaling events involved in bladder cancer biology [19]. Urothelial carcinomas are genetically complex with various oncogenic drivers, numerous mutations within a single tumor, copy number alterations, gene fusion transcripts, and cytogenetic aberrations (Figure 1). Muscle invasive urothelial carcinomas have more mutations, chromosomal aberrations, and aneuploidy than the non-invasive tumors, however, there are common genes implicated in the pathogenesis of both types.

Heat shock proteins (Hsp) are over-expressed in both non-muscle invasive and muscle invasive bladder cancers [20]. They allow bladder cancer cells to survive and progress despite various sources of cellular stress. The heat shock response prevents cancer cells from undergoing apoptosis, despite an accumulation of genomic mutations, and hostile hypoxic and/or acidotic tumor environments [20]. Several proteins involved in bladder cancer biology are regulated by the Hsp90 chaperone complex, which aids in their stabilization, maintains their protein expression and promotes oncogenesis.

### Hsp90: a signaling hub in urothelial carcinoma biology

#### Structure and function

**Figure 2:**
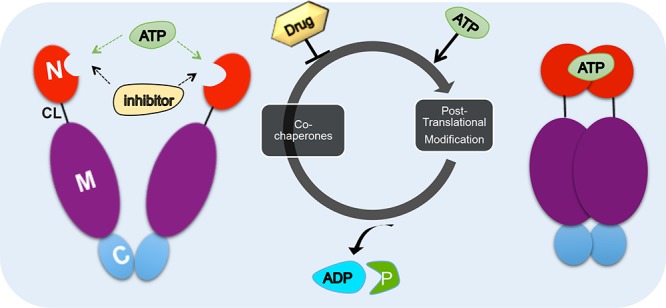
Hsp90 chaperone cycle ATP binding to the N-terminal domain of Hsp90 (red) in an “open” conformation promotes transient dimerization of the N-domains “closed” conformation leading to ATP hydrolysis [38]. The co-chaperones such as Aha1, Cdc37, HOP and p23 and post-translational modification influence the rate of ATP hydrolysis. Domain labeling is as follows: N, N-domain (red); CL, charged linker (black); M, M-domain (purple); C, C-domain (blue).

Hsp90 plays an important role in urothelial carcinoma biology, as well as in carcinogenesis of other tumors, by its function as a molecular chaperone that cancer cells utilize to protect over-expressed or mutated oncoproteins from misfolding and degradation [21–25]. Proteins chaperoned by Hsp90, also known as clients, control numerous cellular processes that support tumor growth and metastasis, including signal transduction, angiogenesis, anti-apoptotic pathways and tumor invasion [26]. Hsp90 is a homo-dimeric protein that comprises of three domains: i) the N-terminal domain, containing nucleotide, drug and co-chaperone (proteins that regulate Hsp90 function) binding sites; ii) the middle (M) domain, which provides binding motif for client proteins and other co-chaperones, and iii) the C-terminal domain containing a dimerization motif and binding sites for yet other co-chaperones. An unstructured charged-linker region connects N and M domains and, therefore, provides conformational flexibility to the Hsp90 protein [27–37] (Figure 2). Hsp90 function is coupled to its ATPase activity [38] and this, in turn, provides conformational cycle that is “fine-tuned” by co-chaperones and post-translational modifications such phosphorylation, acetylation, ubiquitination, oxidation, methylation, *S*-nitrosylation and SUMOylation [39–44] (Figure 2). Clinically evaluated Hsp90 inhibitors disrupt the chaperone cycle by occupying the nucleotide-binding pocket in the N-domain, therefore, inhibiting the ATPase activity [32, 45]. As a result, Hsp90-dependent client proteins are ubiquitinated and degraded in the proteasome [43, 46–48].

#### Protein clients

**Figure 3:**
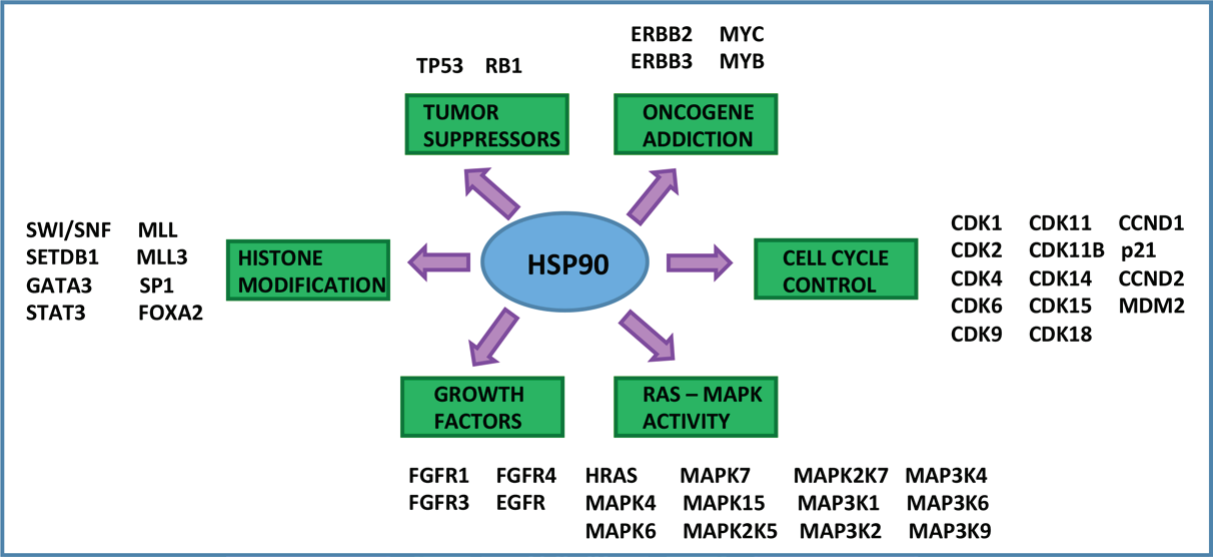
Hsp90 is a central hub to bladder cancer signaling Hsp90 is a critical signaling hub in the etiopathogenesis of urothelial carcinoma. Hsp90 clients include tumor suppressors, oncogenes, growth factors, cell cycle regulators, histone modifying enzymes, and signal transducers [21, 49–55, 57, 58, 60–62]. All of the listed genes are subject to mutation, gene amplification, or deletion in urothelial carcinoma and are Hsp90 client proteins.

Several Hsp90 client proteins act as drivers of urothelial carcinoma, and thus inhibitors or modulators of Hsp90 function may impede urothelial carcinoma pathogenesis (Figures 1 and 3). A recent comprehensive study, by the Cancer Genome Atlas Project, identified novel protein determinants involved in urothelial carcinoma including: *tumor suppressors* (TP53, Rb) [49, 50], *oncogenes* (ErbB2/HER2, ErbB3, Myc) [49, 51, 52], *cell cycle regulatory proteins* (by Hsp90 and Cdc37 co-chaperone, cyclins and cyclin-dependent kinases, including Cdk2) [53, 54], *Ras-MAPK pathway proteins* (HRas and multiple MAP kinases) [55], *mTOR pathway* components (Akt) [56], *growth factor receptors* (EGFR, *FGFR1/3/4*) [57–59] and *transcriptional regulators* which include proteins involved in histone modification (SWI/SNF complex members), STAT3, MLL/3, SP1 and FOXA2 [60–62] (Figure 3).

### Hsp90 and oncogenic signaling pathways in urothelial carcinoma pathogenesis

Urothelial carcinomas have somatic mutations of tumor suppressor genes, which include, but are not limited to TP53, RB1, PTEN, TSC1, and p16, and activation of oncogenic drivers [60, 63] (Figure 1). While mutations in tumor suppressor genes allow for uncontrolled cell proliferation and cell cycle progression, the driver mutation present in many urothelial carcinomas is mutation or gene fusion of the fibroblast growth factor receptor 3 (FGFR3) [58].

#### Fibroblast growth factor signaling

Fibroblast growth factor receptor 3 (FGFR3) is a tyrosine kinase receptor and key regulator of cellular growth and differentiation. FGFR3 depends on Hsp90 for its stability and function and treatment with Hsp90 inhibitors target FGFR3 for degradation [58]. FGFR3 is shown to be over-expressed in 75 percent of non-muscle invasive bladder cancers. It is upregulated by multiple mechanisms in bladder cancer cells: 1) somatic mutation, 2) over-expression of the wild-type protein, or 3) gene fusion with transforming acid coiled coil (TACC3) or BAI-1 associated protein 2-like 1 (BAI1AP2L1) partners [64]. FGFR3-TACC3 or FGFR3-BAI1AP2L1 gene fusions can serve as the drivers of oncogenesis in bladder cancer cells, especially in non-muscle invasive bladder cancers, as they activate numerous downstream signaling pathways, including PI3K-Akt-mTOR, Ras, MAP kinases, STATs, and phospholipase-Cγ [65].

#### Cell cycle deregulation

Loss of cell cycle control is characteristic of carcinogenesis in urothelial carcinoma, as in many other cancers, due to the inactivation of cell cycle-regulating tumor suppressor genes (including TP53, RB, ATM), mutation of cell cycle progression regulators (CDKN1A, CDKN2A, CCND1, and CCNE1), or activating mutations of genes promoting cell cycle progression (including MDM2 and E2F3). Inactivation of the cell cycle regulator p53 results in G1 to S cell cycle progression and uncontrolled cell growth in urothelial carcinomas. p53 is inactivated in muscle invasive bladder cancers to a greater extent than non-muscle invasive bladder cancers (24 versus 76 percent) [17, 60]. Wild type p53 is also a known client of Hsp90, and interacts with Hsp90 through its DNA binding domain [66]. Hsp90 stabilizes mutant p53 through inhibiting the proto-oncogene MDM2 to function as an E3 ubiquitin protein ligase [67, 68]. Although many urothelial carcinomas exhibit p53 inactivation, Hsp90 inhibitors can still promote growth arrest. In lymphocytic leukaemia cells, inhibition of Hsp90 was found to have opposing effects on wild-type and mutant p53 proteins, with stabilization and increased expression of the wild-type protein [69]. Upregulation of p53 results in increased p21 expression, which induces cell cycle arrest at the G1 to S transition point [69]. Hsp90 inhibition induces apoptosis through p53-dependent induction, mediated by PUMA and Bax [68]. Inactivation of the retinoblastoma (RB) tumor suppressor gene is present in approximately 50 percent of high grade and muscle-invasive urothelial carcinomas [70]. Additionally, urothelial carcinomas are the most common carcinomas in survivors with RB cancer [71]. RB loss alone cannot accelerate urothelial proliferation, but inhibits p53. In an animal model for urothelial carcinoma, mice deficient in both RB and P53, but not either tumor suppressor gene alone, were the most sensitive to carcinogen exposure. Growth inhibition induced by Hsp90 inhibitors, occurs through G1 to S phase arrest through reducing RB phosphorylation. RB inhibition reduces expression of cyclin-associated kinases D and E, to prevent cell cycle progression. This was supported by the absence of growth arrest in cells lacking RB, compared to wild-type RB expression [72].

P16 (CDKN2) is a tumor suppressor gene encoded on chromosome 9p21, a region frequently deleted in urothelial carcinomas. P16 arrests the cell cycle through inhibition of cyclin dependent kinases (CDK) 4 and 6. CDK4 and CDK6 (both are Hsp90 clients) phosphorylate RB to stimulate G1 to S phase transition in the cell cycle, therefore, loss of p16 results in uninhibited cell growth [73, 74]. Loss of p16 secondary to deletion occurs in 54 percent of urothelial carcinomas. A study of minimally invasive (T1a) urothelial carcinomas identified that loss of p16 was associated with a reduction in progression-free survival, but did not affect recurrence rates. The loss of p16 was identified as an independent predictor of tumor progression at a given tumor stage and grade [74]. Unlike invasive urothelial carcinomas, p16INK4a is over-expressed in urothelial carcinoma *in situ*, which is secondary to enhanced Ras/MAPK signaling [75].

#### DNA repair

Cells have evolved DNA repair mechanisms essential for the removal of damaged DNA caused by several endogenous (hydrolysis, reactive oxygen species, alkylation, DNA mismatches, insertions or deletions, strand breaks) and exogenous (ultraviolet light, ionizing radiation, chemotherapeutic drugs) sources. As tobacco smoking is considered a risk factor for bladder cancer it is also a source of carcinogens that damage DNA in urothelial cells [76]. Whole-exome sequencing studies identified somatic mutations in genes related to DNA repair pathways including *P53, KDM6A, ATM, ERCC2, FANCD2, PALB2, BAP1, BRCA1* and *-2*) [77, 78]. Interestingly, these mutations seem to be correlated with recurrence-free survival in patients with muscle invasive bladder cancer [77]. As more studies identify alterations in the DNA repair mechanism, poly (ADP-ribose) polymerase (PARP) inhibitors combined with DNA damaging agents may be a good therapeutic strategy in some patients [78].

#### PI3K-Akt-mTOR activation

Activation of the phosphoinositide-3-kinase (PI3K)-Akt-mTOR (mammalian target of rapamycin) pathway occurs in urothelial carcinomas, particularly in muscle-invasive tumors. Its activation is associated with reduced survival and tumor progression [79]. This usually occurs secondary to PTEN (phosphatase and tensin homolog) deletion, which results in increased activation of Akt by uninhibited PI3K activity. Akt activation results in mTORC2 (mTOR complex 2) activation and subsequent changes in cytoskeletal dynamics. It also promotes mTORC1 (mTOR complex 1) activation through inhibition of a negative regulator, the tuberous sclerosis complex (TSC1 and TSC2). TSC1/TSC2 complex inhibits Rheb (Ras homolog enriched in brain) GTPase, which activates mTORC1. mTORC1 stimulates cellular growth through regulation of protein translation by its targets S6 kinase and 4E-BP1. Akt, which activates both mTORC1 and 2, is an Hsp90 client [56].

PTEN, a tumor suppressor gene that negatively regulates activation of the PI3K/Akt/mTOR pathway, is deficient in over 50 percent of urothelial carcinomas due to loss of heterozygosity (LOH). Microdeletions at the 10q23 locus is often the source of PTEN LOH. Reduced PTEN levels leads to hyperactive Akt through PDK1 (3-phosphoinositide-dependent kinase 1)-mediated phosphorylation. Hsp90 stabilizes Akt and under Hsp90 inhibition, Akt is rapidly ubiquitinated, greatly shortening its half-life (to one-third) within treated cells [56].

#### Epithelial-mesenchymal transition

There are several signaling pathways that cooperate to induce epithelial-to-mesenchymal transition (EMT) in cancer [80]. One of them is the activation of the Ras-MAPK pathway [18]. A low level of Ras-MAPK activation is present in non-invasive urothelial carcinomas, while hyperactivation occurs in high-grade tumors [18]. The Ras-MAPK pathway is activated by receptor tyrosine kinases, as well as by fibroblast growth factor signaling. In addition, Src signaling (c-Src depends on Hsp90 for its maturation) stimulates EMT through cytoskeletal remodeling and inhibiting formation of adherens junctions [81].

#### Epigenetic control and transcriptional regulation

Reduced histone modification with inactivation of acetyltransferases, methyltransferases, deubiquitinases, and demethylating enzymes occurs in 89 percent of urothelial carcinomas [60]. There are numerous chromatin modifying or remodeling enzymes subject to frequent mutations in urothelial carcinoma, which include BAP1, UTX, MLL, NCOR1, ARID1A, CHD1 and 6, CREBBp, EP300, and the SWI/SNF complex [78, 82]. Mutation causes inactivation of chromatin modifying enzymes in over 60 percent of urothelial carcinomas, with the most common mutations within members of the SWI/SNF complex [60]. The SWI/SNF complex maintains an environment of tumor suppression, and is frequently lost in urothelial carcinomas [83, 84].

### Current and investigational treatments for urothelial carcinoma

Treatment of metastatic urothelial carcinoma remains a major challenge, with no improvement in overall survival achieved in the past 20 years [85]. Patients with advanced urothelial carcinoma have a poor overall survival of less than 30 percent at 5 years post-diagnosis [86]. Yet, chemotherapy still provides benefit over cystectomy, alone [87, 88]. Generally, 70 percent of patients with metastatic urothelial carcinoma respond to systemic chemotherapy, however more than 90 percent of them relapse and eventually die from the disease [89].

Although several molecular targeted therapies for urothelial carcinoma are under investigation, no new agents have been approved by the Food and Drug Administration (FDA) in the past 3 decades. The only FDA approved therapies to date for muscle invasive bladder cancer are cisplatin, doxorubicin hydrochloride, and gemcitabine hydrochloride, all of which are cytotoxic chemotherapies. Non-muscle invasive bladder cancers are primarily treated with transurethral resection and intravesical therapies.

#### Intravesical therapies

Currently, there are several intravesical therapies for the treatment of non-invasive urothelial carcinomas, including Mitomycin C, epirubicin, doxorubicin, and instillation of live attenuated *Mycobacterium bovis* bacillus Calmette-Guérin (BCG) [90]. BCG reduces the risk of progression and recurrence and is used to treat patients with carcinoma *in situ* as well as non-invasive urothelial carcinomas (T1a tumors) [91–96]. Despite the success of intravesical treatments, disease progression and recurrence are common, with a lifetime recurrence risk of 88 percent in some studies [97]. Additionally, with the need for frequent surveillance, repeat biopsies, treatment of recurrence, and management of complications, the costs associated with treatment of urothelial carcinoma is very high, often exceeding $150, 000 over the patient's lifetime [6].

#### Chemotherapies

The alkylating agent cisplatin, the topoisomerase II inhibitor, doxorubicin hydrochloride and the nucleoside analog gemcitabine hydrochloride, all are non-specific blockers of DNA synthesis in proliferating cells [98–100]. There are many toxicities associated with these drugs, which include but are not limited to bone marrow suppression with pancytopenia, peripheral edema and capillary leak syndrome, pulmonary toxicity including respiratory distress syndrome and pulmonary fibrosis, gastrointestinal toxicities with weight loss, hepatotoxicity that can lead to liver failure, renal toxicity and hemolytic-uremic syndrome and infections [101, 102].

One of the most commonly used combination regimens for advanced bladder cancer, includes methotrexate, vinblastine, doxorubicin, and cisplatin, only leads to a complete response in 1 of 4 patients [103]. Cisplatin is a chemotherapy agent that binds to purine DNA bases causing cross-linking and triggering DNA damage response [104]. Overcoming the DNA damage response can lead to drug resistance [104, 105]. This high rate of treatment failure has been attributed, in part, to the development of cisplatin resistance, which occurs through multiple mechanisms, such as reduced cisplatin binding to DNA (pre-target resistance), reduced formation of DNA-cisplatin adducts (on-target resistance), altered DNA damage response (post-target resistance), and influenced signaling pathways that reduce the DNA damage response (off-target resistance) [104, 106]. More recently, Choi et al. identified a p53-like subtype of invasive bladder cancer associated with cisplatin-based chemotherapy resistance to apoptosis [107]. In addition to resistance, many patients with urothelial carcinoma have impaired renal function, and cisplatin is nephrotoxic [108]. Therefore, individuals with impaired renal function are not candidates for cisplatin treatment [109]. Given the resistance and toxicity of cisplatin, alternative strategies to sensitize bladder cancer cells to chemotherapy and allow decreased dose of cisplatin, while increasing the response and durability, are being explored.

Several clinical trials are ongoing for the treatment of advanced, unresectable, or metastatic urothelial carcinoma (Table 1). These include new chemotherapeutics targeting the DNA damage response or histone deacetylases, inhibitors of the mammalian target of rapamycin (mTOR), tyrosine kinase inhibitors (TKIs), HER2 blockade, anti-angiogenic therapies, and immunotherapies. Immunotherapy approaches include BCG [110], cytokine-based treatments (recombinant IL-2 or IL-7) to elicit cellular immune responses [111], or dendritic cell vaccines [112].

**Table 1:** FDA-approved and investigational therapies for urothelial carcinoma

Drug category	Drug urothelial carcinoma	FDA approved or clinical trial phase	Type / stage of cancer
**Chemotherapy**	Cisplatin (alkylating agent)	Approved	T4b and metastatic urothelial carcinoma
	Doxorubicin hydrochloride (topoisomerase II inhibitor)	Approved	Stage IV and recurrent bladder cancer
	Gemcitabine hydrochloride	Approved	Advanced bladder cancer
	5-fluoro-2′-deoxcytidine + tetrahydrouridine	Phase II	Advanced bladder cancer
	Eribulin mesylate (E7389)	Phase I / II	Locally advanced or metastatic bladder cancer
	Veliparib / ABT-888	Phase I	Non-resectable or metastatic
	Romidepsin (histone deacetylase inhibition)	Phase I	Advanced urothelial carcinoma
**Anti-tumor immunity**	rhIL-7 vaccine	Phase II	Metastatic urothelial carcinoma
	DC205-NY-ESO-1 fusion protein vaccine +/− sirolimus	Phase I	Metastatic urothelial carcinoma
	Ad/HER2/Neu dendritic cell vaccine	Phase I	T3a and above HER2+ bladder cancer
	Bacillus Calmette-Guerin + PANVAC	Phase II	Non-muscle invasive bladder cancer
	ALT-801 (IL-2 recombinant fusion protein) post-chemotherapy with cisplatin and gemcitabine	Phase I / II	Advanced stage muscle-invasive bladder cancer
**mTOR inhibitors**	ABI-009 (nab-rapamycin)	Phase I / II	Advanced non-muscle invasive bladder cancer
	Sirolimus, post-chemotherapy with cisplatin and gemcitabine hydrochloride	Phase I / II	T2 to T4 tumors
**Anti-angiogenic therapy**	Cabozantinib	Phase II	Advanced stage bladder cancer
	Bevacizumab, post-chemotherapy with gemcitabine hydrochloride + cisplatin	Phase III	Metastatic, unresectable, or locally advanced bladder cancer
	Lenalidomide, post-chemotherapy with gemcitabine hydrochloride and carboplatin	Phase I	Unresectable or metastatic bladder cancer
**Tyrosine kinase inhibitors**	Afatinib	Phase II	Ureteral cancer, stage III, stage IV urothelial carcinoma
	Dovitinib	Phase II	BCG-refactory urothelial carcinoma, FGFR3-mutated urothelial carcinoma
	Erlotinib	Phase II	Stage I, II, III and recurrent urothelial carcinoma
	Gefitinib	Phase II (completed)	Locally advanced and metastatic bladder cancer
	Pazopanib	Phase II (completed)	Locally advanced and metastatic bladder cancer
	Sorafenib	Phase II (completed)	Locally advanced and metastatic bladder cancer
	Sunitinib	Phase II	BCG-refactory bladder cancer
**HER2 blockade**	Afatinib (targets EGF and HER2)	Phase II	Refactory bladder cancer
	MGAH22, a human chimeric antibody against HER2	Phase I	HER2 positive bladder cancer

#### New chemotherapies

Additional chemotherapies under early stage investigation for urothelial carcinoma include the combination of 5-fluoro-2′-deoxycytidine and tetrahydrouridine (antimetabolites), eribulin mesylate (an inhibitor of microtubule dynamics), veliparib (an inhibitor of PARP 1 and 2, which prevents DNA repair within cancer cells to enhance chemoradiosensitivity), and ropidepsin (a histone deacetylase inhibitor). As many bladder cancer tumors possess cells with somatic mutations in genes associated with DNA repair, they should be especially sensitive to chemotherapeutic drugs that promote DNA damage [78]. Chemotherapy remains the first-line therapy for metastatic urothelial carcinoma, but several factors make some patients poor treatment candidates, including poor performance status, renal insufficiency, neuropathy, ototoxicity, and heart failure (New York Heart Association grade 3 and above) [113].

#### Tyrosine kinase inhibitors

Tyrosine kinase inhibitors (TKIs) evaluated in clinical studies for urothelial carcinoma include afatinib, erlotinib, dovitinib, sunitinib, gefitinib, pazopanib, and sorafenib. Erlotinib primarily targets the epidermal growth factor receptor (EGFR), and was shown to have optimal use in the neoadjuvant setting, resulting in downstaging of the tumor prior to surgery [114]. Sunitinib is an oral, small-molecule, multi-targeted tyrosine kinase inhibitor, with significant activity against VEGFR, PDGFR, stem cell factor receptor, fms-like tyrosine kinase 3 (Flt3) and the tyrosine kinase receptor encoded by the RET proto-oncogene. A double-blind, randomized, phase 2 trial of maintenance sunitinib versus placebo was tested in MIBC patients who have previously undergone chemotherapy, and was found to have anti-tumor activity [115], however maintenance sunitinib did not appear to improve the 6-month progression rate [116], therefore, it is not an ideal second-line option for patients unable to tolerate cisplatin-based chemotherapy regimens [113]. Sorafenib is an oral, multi-kinase inhibitor which blocks tumor cell proliferation by targeting the Ras/Raf/ERK pathway at the level of Raf kinase, and blocks angiogenesis by targeting the VEGFR and PDGFR families. It was found to have insufficient activity as a first-line treatment for advanced urothelial carcinoma [117]. Pazopanib is an oral multi-kinase angiogenesis inhibitor that targets VEGFR, PDGFR and stem cell receptor factor. Synergistic efficacy of pazopanib with docetaxel was demonstrated in docetaxel-resistant bladder cancer cells [118]. However, in a phase 2 trial, Pazopanib did not show significant activity against metastatic urothelial carcinoma [119]. Gefitinib is an oral, small-molecule inhibitor of the intracellular domain of EGFR [120]. Gefitinib was found to have a growth-inhibitory and anti-invasive effect in urothelial carcinoma cell lines [120]. However, gefitinib demonstrated minimal anti-tumor activity in patients with metastatic urothelial carcinoma in patients with prior chemotherapy and is ineffective as a second-line agent for urothelial carcinoma or in combination therapy with cisplatin and gemcitabine [121]. Dovitinib (TKI258) is another TKI with promising pre-clinical data, significantly retarding the growth of bladder tumor xenografts *in vitro* and *in vivo* [122], however, it had limited single agent activity in previously treated muscle invasive bladder cancer patients in a phase II clinical study [123]. Lapatinib is a TKI with dual targeting of EGFR and ERBB2, most suitable for metastatic urothelial carcinoma patients with EGFR or ERBB2-overexpressing tumors [124], and can be used post-chemotherapy or in chemoresistant patients [121].

#### Angiogenesis inhibitors

Anti-angiogenic therapies include bevacizumab, aflibercept, lenalidomide, and the anti-angiogenic TKIs (cabozantinib, pazopanib, and sorafenib). Bevacizumab is used in conjunction with cisplatin and gemcitabine as a first-line treatment for metastatic urothelial carcinoma. A recent phase II clinical trial showed an increase in overall survival and had an acceptable toxicity profile [125]. A phase III study is currently ongoing (Gemcitabine Hydrochloride and Cisplatin With or Without Bevacizumab in Treating Patients With Advanced Urinary Tract Cancer (NCT00942331)). Another anti-angiogenic therapy, Aflibercept, is a human recombinant fusion protein that acts as a soluble decoy receptor, also known as a “VEGF Trap” [126]. It contains vascular endothelial growth factor receptor-1 and 2 (VEGFR-1 and 2) fused to the Fc fragment of IgG1, which inhibits VEGF signaling. Aflibercept fusion protein is used after platinum-based chemotherapy, and has limited single-agent activity, based on a phase II clinical study [126].

#### HER-2 blockade

Expression of the HER-2/neu oncogene is associated with tumor invasion and metastatic potential and is expressed on 28 percent of urothelial carcinomas overall, and greater than 50 percent of muscle-invasive tumors [127]. Lapatinib, a tyrosine kinase inhibitor, elicits dual blockade of EGFR and HER-2/neu [128]. Although both of these targets are over-expressed on urothelial carcinomas and play pathogenic roles, treatment with lapatinib as a second-line therapy for metastatic urothelial carcinoma in combination with paclitaxel was poorly-tolerated [129].

#### Mechanistic target of rapamycin (mTOR) inhibitors

Inhibitors of the mTOR have demonstrated anti-tumor activity alone and in combination with chemotherapy, as mTOR inhibition enhances chemosensitivity of urothelial carcinoma cells [130]. mTOR inhibitors induce cell cycle arrest at the G0 to G1 growth phase and inhibit VEGF production [131]. Everolimus (also known as RAD001) has synergistic activity with cisplatin, and provides anti-tumor activity in a subset of patients with muscle invasive bladder cancer [132, 133]. Everolimus evoked an anti-angiogenic response, but requires functional PTEN, as PTEN loss was associated with resistance to everolimus and other mTOR inhibitors [134, 135]. Long-term rapamycin (also known as Sirolimus) treatment reduced the incidence of urothelial carcinoma in renal transplant recipients [136], who are at increased risk of malignancy due to a reduced ability to elicit anti-tumor immune responses, however the protection against incidence and recurrence was not 100 percent [137]. Temsirolimus was tested in a phase II clinical trial after failure of platinum based therapy for muscle invasive bladder cancer, but yielded poor treatment responses [138].

#### Immunotherapies

Immunotherapies for urothelial carcinoma include instillation of live but attenuated BCG, dendritic cell-based vaccines, recombinant IL-7, and recombinant IL-2 fusion protein. These are all aimed at improving anti-tumor immune responses. BCG elicits production of hydrogen peroxide, superoxide, and free radical oxygen, promoting oxidative stress within urothelial carcinoma cells [139]. Oxidative stress promotes DNA damage and lipid peroxidation, which leads to tumor cell death and release of the “danger signal” HMGB1 (high-mobility group protein 1) that stimulates the innate immune response [140]. AdCD40L adenoviral vaccine is used to upregulate CD40 ligand to improve anti-tumor immune responses as a neoadjuvant treatment for muscle invasive bladder cancer undergoing cystectomy, and has successful gene transfer causing enhanced immune activation [141]. A completed phase I/II trial demonstrated safety and induction of an immune response (identified by increased numbers of IFNγ-producing T lymphocytes) [141], and is currently under evaluation in a larger phase II trial for evaluation of efficacy. NY-ESO-1 vaccine, in conjunction with BCG and sargramostim is under investigation in muscle invasive bladder cancer patients post-cystectomy that have tumors that express NY-ESO-1 or LAGE-1 antigens (expressed in nearly 50 percent of muscle invasive bladder cancers) [142]. Immune responses against these antigens were present in patients having received the vaccination for advanced urothelial carcinoma [143]. CDX-1307, a monoclonal antibody that targets the mannose receptor and beta-hCG (human chorionic gonadotropin), is also under evaluation in clinical trials [144]. Increased numbers of CD8+ cytotoxic lymphocytes infiltrating the tumor is associated with beneficial anti-tumor immune responses and serves as a good prognostic indicator [145]. These current and investigational treatments for urothelial carcinoma are summarized in Table 2.

**Table 2:** Preclinical studies of Hsp90 inhibitors in bladder cancer

Hsp90 inhibitor	Combination therapy	Model system	Outcome	Mechanism	Reference
**17-AAG**	Cisplatin	JTC-30 (low grade papillary), RT4 (grade 1), KK47 (grade 1), 5637 (grade 2), 1376 (grade 3), and T24 (grade 3) bladder cancer cell lines	Synergistic reduction in cell survival	↓ Activation of Erk1/2, Akt, PI3K ↑ Apoptosis ↓ Cell cycle progression	[160, 174]
**17-AAG**	Docetaxel	RT4 (grade 1), KK47 (grade 1), 5637 (grade 2), 1376 (grade 3), and T24 (grade 3) bladder cancer cell lines	Synergistic reduction in cell survival	↑ Apoptosis ↓ Cell cycle progression	[160]
**17-AAG**	Gemcitabine	RT4 (grade 1), KK47 (grade 1), 5637 (grade 2), 1376 (grade 3), and T24 (grade 3) bladder cancer cell lines	Synergistic reduction in cell survival	↓ Chk1 ↑ Apoptosis ↓ Cell cycle progression	[160]
**17-AAG**	Pifitrhrin-μ	RT4 (grade 1), KK47 (grade 1), 5637 (grade 2), 1376 (grade 3), and T24 (grade 3) bladder cancer cell lines	Synergistic reduction in cell survival	↓ p-Akt, p-Bad ↑ Apoptosis	[160]
**17-AAG**	Cisplatin + radiotherapy	5637 (grade 2), T24 (grade 3), and UM-UC-3 bladder cancer cell lines	Chemoradio-sensitization	Inactivation of anti-apoptotic proteins erbB2, Akt, NF-kB	[161]
**17-AAG**	Cisplatin	SCID xenografts with 5637 bladder cancer cells Bladder cancer initiating cell xenograft model	Increased reduction in tumor size compared to cisplatin alone No treatment related death or weight loss	Inactivation of anti-apoptotic proteins erbB2, Akt, NF-kB	[174] [161]
**17-DMAG**	Cisplatin + radiotherapy	5637 (grade 2), T24 (grade 3), and UM-UC-3 bladder cancer cell lines	Chemoradio-sensitization	Inactivation of Akt and Erk ↓ Survival of bladder cancer cells	[161]

### Hsp90 inhibitors in treating urothelial carcinoma

One promising target to enhance clinical responses is treatment with Hsp90 inhibitors. There are currently sixteen Hsp90 inhibitors evaluated in clinical trials for numerous hematopoietic and solid malignancies [22]. These include melanoma, small cell lung cancer, non-small cell lung cancer, breast cancer, gastrointestinal stromal tumor, gastric cancer, colon adenocarcinoma, ovarian cancer, primary peritoneal cancer in women, fallopian tube cancer, prostate adenocarcinoma, leukemia, lymphoma, myeloproliferative disorders, and myelodysplastic syndromes [146–148]. Several Hsp90 inhibitors have demonstrated safety, while the second generation Hsp90 inhibitor, ganetespib, is currently in phase III clinical trials (for non-small cell lung cancer) and already has shown manageable side-effects in phase II clinical trials [149]. We do not expect that molecular targeted therapy will replace current treatments for urothelial carcinoma, rather it may be used adjunctively with other therapies to heighten the clinical response and reduce residual disease or recurrence.

As numerous client proteins participate directly in the pathogenesis of urothelial carcinoma, Hsp90 could be an ideal target [21] (Figures 1 and 3). Targeting Hsp90 also inhibits pathways involved in tumor invasion and metastasis, by affecting cellular migration and angiogenesis [150]. For instance, Hsp90 blockade by geldanamycin in bladder cancer cells was shown to inhibit signaling by the hepatocyte growth factor and its target oncogene c-Met, which participates in tumor cell migration by disruption of extracellular matrix components [150]. Over-expression of c-Met is associated with poor prognosis in urothelial carcinoma and can be blocked by Hsp90 inhibitors [151, 152]. Hsp90 inhibition also inhibits hypoxia-inducible factor signaling, another factor associated with poor prognosis in urothelial carcinoma due to induction of angiogenesis promoted by vascular endothelial growth factor signaling [153, 154].

In urothelial carcinoma, no distinct correlation exists between Hsp90 protein levels and grade or pathologic stage of the cancer [155, 156], yet its inhibition results in destabilization of ErbB2, NF-κB, and phosphorylated Akt [157]. These proteins are Hsp90 clients [158, 159], and their collective inhibition prevents the usual deregulated cell growth and desensitization to pro-apoptotic signals [160, 161].

More recently, bladder cancer cell lines with FGFR3 somatic mutation, FGFR3-TACC3 and FGFR3-BAI1AP2L1 gene fusions were all found to be sensitive to Hsp90 inhibition. Urothelial carcinoma cells with FGFR3 mutations that are insensitive to pan-FGFR inhibitors (those containing the FGFR3^S249C^ or FGFR3^Y375C^ mutations) were found to be sensitive to Hsp90 inhibitors [162]. While both ganetespib and an FGFR inhibitor promoted regression of bladder cancer xenografts harboring the FGFR3-TACC3 fusion, combination of the two agents led to a further, significant reduction in tumor volume [162]. Thus, the use of HSP90 inhibitors alone or in combination with targeted therapies may serve as a therapeutic strategy for genetically defined bladder cancers.

### The effects of Hsp90 inhibitors in combination with chemotherapy in treating urothelial carcinoma

In the etiopathogenesis of bladder cancer and other solid tumors, there is increased expression of Hsp90 [163]. Hsp90 inhibition alone has cytostatic effects in bladder cancer cell lines *in vitro* and it also provides synergistic effects with various chemotherapy drugs [160], including those used in the clinic for urothelial carcinoma treatment. Treatment of head and neck cancer cells with Hsp90 inhibitor 17-AAG was shown to activate apoptosis by restoring wild type p53 function through the disruption of p53 interaction with its negative regulator murine double minute proteins X (MDMX) [164]. When cells were treated with a combination of Hsp90 inhibitor 17-AAG and cisplatin there was a more prominent apoptotic effect *in vitro*, tumor growth inhibition *in vivo* and restoration of wild type p53 levels. Hsp90 inhibitors provide synergism with cisplatin, and likewise, cisplatin improves clinical responses to Hsp90 inhibitors by preventing the compensatory heat shock response due to Hsp90 inhibition [165]. The first generation Hsp90 inhibitor 17-AAG was found to have synergistic anti-tumor activity when combined with cisplatin and/or gemcitabine in treating refractory and metastatic solid tumors, including urothelial carcinoma in patients with unresectable or metastatic disease [166]. However, the ability to escalate the dose of 17-AAG when used with chemotherapy drugs was limited, due to hematologic toxicities [166]. Second generation Hsp90 inhibitor ganetespib potentiated the cytotoxicity of doxorubicin and improved shrinkage of metastatic lesions when used in combination therapy in a study of triple negative breast cancer [167]. This could be due to induction of DNA damage, resulting in mitotic arrest and enhanced apoptosis in cancer cells. Thus, Hsp90 inhibitors have been investigated in combinatorial use for all current FDA-approved treatments for urothelial carcinoma and show promise, as they provide synergy with chemotherapy.

Hsp90 inhibitors are also radio-sensitizing compounds [101, 168]. They prevent DNA damage response after exposure to radiation to induce apoptosis in treated cells, providing synergism with radiation therapy [169, 170]. Radiation therapy is used adjunctively with multi-agent chemotherapy regimens in bladder-sparing protocols and in those with muscle-invasive disease [171] and, thus, Hsp90 inhibitors may provide added benefit. In acting synergistically with chemotherapy, Hsp90 inhibitors may provide an alternative to adjunctive radiotherapy and may reduce morbidities, as a standard radiation dose (54 – 64 Gray) promotes radiation cystitis [161].

Therefore, Hsp90 inhibitors can sensitize tumor cells to chemotherapy or radiotherapy [172, 173]. They can also provide anti-tumorigenic defense at low non-cytocidal doses when combined with chemoradiation [174]. CD44-expressing tumor-initiating cells in bladder cancer have been found to confer resistance to cisplatin compared to CD44- negative cells [174]. Combination of Hsp90 inhibition and chemoradiotherapy potentiated significant apoptosis of Grade III T24 bladder cancer cells compared to cells received single treatments. This elevated sensitization by 17-AAG may overcome cisplatin resistance [161, 174]. One mechanism of chemoradiosensitization by Hsp90 inhibitors is through reducing expression of the oncoproteins ErbB2 and NF-kB as they participate in resistance to chemoradiotherapy [175] and are Hsp90 clients [21].

### Resistance to Hsp90 inhibitors

Hsp90 inhibition activates the heat shock response, which has been shown to limit efficacy of Hsp90 inhibitors in cancer therapy [176–179]. Of note, Hsp27, 40, and 70 are upregulated when Hsp90 is inhibited [180]. Hsp27 prevents protein aggregation of Hsp90 client proteins; Hsp40 acts as an ATPase modulator for both Hsp90 and Hsp70 [180, 181]. Blocking the compensatory heat shock response by Hsp27, 40, and 70 can reduce resistance to Hsp90 inhibitors [180]. Increased expression of heat shock proteins in urothelial carcinoma is associated with poor prognosis and treatment resistance [20, 182]. Multi-targeted Hsp inhibition increases apoptosis, induces G2/M cell cycle arrest, and inhibits autophagy in cancer cells [160, 179, 183]. Inhibition of mTOR inhibits upregulation of heat shock factor (HSF) in response to Hsp90 inhibition, and would also serve as a means to reduce resistance [184].

Further, it was noted that bladder cancer cell lines which express UGT1A (UDP-glucuronosyltransferase) enzymes were insensitive to resorcinol-based HSP90 inhibitors such as ganetespib and NVP-AUY922 (both UGT1A substrates), but sensitive to the ansamycin-based HSP90 inhibitors suggesting that intratumoral metabolism plays a role in drug resistance. Thus, UGT1A expression in bladder cancer may represent a predictive biomarker for what appears to be the most clinically advanced HSP90 inhibitors [162].

Hsp90 has a second drug-binding site in the C-domain [185] and coumarin derivative antibiotics target this site without activating HSF [186, 187]. This is unlike the effect linked to the Hsp90 N-domain inhibitors. Existing data strongly supports further medicinal chemistry optimization and preclinical evaluation of C-terminal Hsp90 inhibitors in urothelial carcinoma [188].

In summary, HSP90 inhibition provides novel opportunities for targeting common pathways evolved in urothelial carcinoma of the bladder, increasing sensitivities to known chemotherapeutic agents, and potentially optimizing sensitivity to radiation therapy.

## CONCLUSION

Urothelial carcinoma is driven largely by loss of the tumor suppressor genes p53, PTEN, RB, and p16, requiring restoration of loss of function versus gain of function, which can more easily be targeted. There are also limited options available for patients with advanced disease who progress on or are ineligible for chemotherapy, resulting in poor outcomes. Our increased understanding of tumor biology and molecular pathways involved in urothelial carcinoma has allowed us to explore unique treatment targets. The inhibitors of the molecular chaperone Hsp90 have shown promise in clinical trials for other epithelial malignancies. Combination of Hsp90 inhibitors with chemotherapeutic agents provides better response rates in urothelial carcinoma *in vitro*. Further studies with new-generation Hsp90 inhibitors in the treatment of urothelial carcinomas will unravel the optimal combinational therapy, with a possible decrease in drug resistance. This may ultimately provide a long-term survival benefit and disease remission.
